# A Case of Pyrexia of Unknown Origin Diagnosed as Hemophagocytic Lymphohistiocytosis

**DOI:** 10.7759/cureus.53553

**Published:** 2024-02-04

**Authors:** Jagannath S Dhadwad, Ramiz S Kadiwala, Kunal K Modi, Prince R Yadav, Subashini P Vadivel

**Affiliations:** 1 General Medicine, Dr. D.Y. Patil Medical College, Hospital and Research Centre, Dr. D.Y. Patil Vidyapeeth, Pune, IND

**Keywords:** pseudomonas aeruginosa, multi organ dysfunction, undiagnosed fever, pyrexia of unknown origin, hyperferritinemia, autoimmune, hlh, hemophagocytic lymphohistiocytosis

## Abstract

Hemophagocytic lymphohistiocytosis (HLH) is a rare disease that is even rarer in the adult population. It requires a high degree of suspicion from the treating physician, and if diagnosed early, patients might have a survival benefit from this highly fatal condition. HLH is a disorder of immune regulation where the hyperactivity of cytokines attacks different cells, which leads to multiple organ dysfunctions. Varying presentations and similarities with other diseases make diagnosis difficult. Familial HLH is commonly seen in the pediatric population, while acquired or secondary HLH is seen in adults. Secondary HLH is commonly triggered by neoplasms, infections, rheumatological diseases, and other autoimmune diseases. Here is a case of HLH that presented as chronic undiagnosed fever. In this case report, we have discussed in detail this disease, its presentation, investigations, treatment, and other important information that will help practicing doctors better diagnose and treat HLH patients.

## Introduction

Hemophagocytic lymphohistiocytosis (HLH) is a syndrome of excessive immune activation that commonly affects infants but can be seen in all age groups [1,2]. Primary HLH is autosomal recessive with an estimated incidence of around 1:50000 live births and seen in pediatric and adolescent populations, while secondary HLH is commonly seen in adults around the age of 50 years [3]. Triggers for secondary HLH are infections that cause immune activation, like Epstein-Barr virus (most common) [4], herpes virus, cytomegalovirus, dengue virus, coronavirus disease 2019 (COVID-19) virus, as well as conditions that cause immune suppression like malignancy, rheumatological conditions, or HIV infection. It is extremely rare to have a gram-negative septicemia-induced HLH. Here, we discuss an interesting case of secondary HLH triggered by *Pseudomonas aeruginosa*.

## Case presentation

A 51-year-old male farmer came with complaints of fever with chills for three months, which increased in severity with time, recorded a peak of 102 ℉, more during evening hours, fever spikes were continuous for two hours, and did not respond to conventional antipyretic medication during that period. Fever was associated with nausea, a loss of appetite, and generalized malaise. Non-productive cough for three months, more in the supine position, is associated with headaches and nausea after cough bouts. He had a history of significant weight loss of 13 kg in the last two months; no history of sore throat, hemoptysis, breathlessness, chest pain, or orthopnea, no history of abdominal pain, vomiting, melena, hematochezia, seizure, neck rigidity, muscle spasms, or abnormal body movements; and no history of burning micturition, bladder incontinence, abdominal distention, or yellowish discoloration of the eye or skin. He was known hypertensive for nine months on tab. bisoprolol 5 mg once daily (OD). He had a history of ischemic heart disease. Percutaneous coronary intervention to the left circumflex artery was done nine months ago, currently on tab. aspirin 75 mg OD, tab. prasugrel 10 mg OD, and tab. rosuvastatin 20 mg OD. He had a history of COVID-19 two years ago and no history of tuberculosis.

On general examination, blood pressure was 90/60 mmHg in the right arm in the supine position. Pulse was 117 bpm, regular, high-volume, no radio-radial or radio-femoral delay, and all peripheral pulses felt. His temperature was 100 ℉ in the right axilla; oxygen saturation (SpO2) was 99% on room air, and his respiratory rate was 18 breaths per minute. Pallor was present, but no icterus, no cyanosis, no clubbing, no palpable superficial lymph nodes, and no edema were noted. Jugular venous pressure was not raised, and no rash or petechiae were seen.

On systemic evaluation, cardiovascular system inspection and palpation were normal. On auscultation, a holosystolic murmur at apex radiating to axilla. On per abdominal examination, the abdomen was soft and non-tender, the umbilicus centralized, with no scar marks or dilated veins, the liver was palpable 5 cm below the right inferior costal margins and the spleen was palpable below the left costal margin and normal in consistency, on abdominal percussion, there was no shifting dullness, and the liver span was 19 cm. Respiratory and central nervous system examinations were normal.

Initial blood investigations are given in Table 1 and Table 2. The chest radiograph was normal, the electrocardiogram was within normal limits, and the peripheral blood smear showed microcytic, hypochromic red blood cells, and moderate thrombocytopenia.

**Table 1:** Day-wise routine investigations with reference ranges. TLC – total leucocyte count, MCV – mean corpuscular volume, ANC – absolute neutrophil count, INR – international normalised ratio, aPTT – activated plasma thromboplastin time, PCV – packed cell volume, RDP – random donor platelets, FFP – fresh frozen plasma

Investigations	Day 1	Day 2	Day 3	Day 4	Day 5	Day 6	Day 8	Reference Range
Hemoglobin	7.40	7.20	6.0	6.8 (1 PCV given)	6.9 (1 PCV given)	7.1	7.4	13.2-16.6 g/dL
TLC	4600	3900	3100	2900	2100	4100	5900	4000-10000/microL
ANC	1900	1800	1400	1300	800	1700	2800	2000-7000/microL
Platelets	64000	59000	45000	44000 (4 RDP given)	31000 (4 RDP given)	66000	81000	150000-410000/microL
MCV	89.10	89.00	91.00	90.40	87.90	87.70	89.20	78.2–97.9 fL
Total Bilirubin	1.91	2.01	2.2	2.3	3.1	2.7	2.1	0.22-1.20 mg/dL
Direct Bilirubin	0.89	1.13	1.2	1.3	2.1	2.0	1.6	<0.5 mg/dL
Aspartate Transaminase	180	199	580	1210	2551	970	440	8-48 U/L
Alanine Transaminase	166	184	545	880	940	590	165	7-55 U/L
Alkaline Phosphatase	290	310	300	350	410	390	310	40-129 U/L
Urea	82	79	102	111	124	91	66	17-49 mg/dL
Serum Creatinine	2.92	2.60	3.3	3.7	3.9	3.1	2.3	0.6-1.35 mg/dL
INR	1.10	-	1.24	2.9 (6 FFP given)	-	2.1	1.40	0.85-1.15
aPTT	27.5	-	29.5	35.5	-	31.1	30.3	21.75-28.70 sec
Serum Sodium	136	134	129	131	130	128	134	136-145 mmol/L
Serum Potassium	4.33	4.10	3.70	3.90	4.40	4.50	4.40	3.50-5.10 mmol/L

**Table 2:** Other important initial investigations with reference ranges. RBCs – red blood cells

Investigations	Day 1	Reference Range
Serum Calcium	8.9	8.5-10.5 mg/dL
Serum Phosphate	3.60	2.5-4.5 mg/dL
Serum Uric Acid	6.30	2.5-7.0 mg/dL
Serum Total Proteins	6.1	6.4-8.3 g/dL
Serum Albumin	3.2	3.5-5.5 g/dL
Serum Globulin	2.9	2.0-3.5 g/dL
Urine routine & microscopy	Protein – 1 +	Protein – absent
	Glucose – absent	Glucose – absent
	Pus cells – 1-2	Pus cells – 0-5/hpf
	RBCs – 2-3	RBCs – 0-2 cells/hpf
	Urinary casts or crystals – absent	Urinary casts or crystals – absent

The patient was started on inj. piperacillin and tazobactam according to renal clearance. Inj. caspofungin was also started because of neutropenia along with other supportive care and nutrition. The patient had continuous fever spikes so inflammatory markers were sent on day two as shown in Table 3. Iron studies showed a very aberrant picture with low iron stores and significantly higher ferritin levels as shown in Table 4. Table 5 summarizes all the special investigations sent till the fourth day of admission.

**Table 3:** Inflammatory markers and further investigations with their reference ranges. ESR – erythrocyte sedimentation rate, CRP – C-reactive protein, LDH – lactate dehydrogenase, GGT – gamma glutamyl-transferase

Investigations	Day 2	Day 3	Reference Range
ESR	51	74	0-20 mm/hr
CRP	185	230	<10 mg/L
LDH	259	-	105-234 IU/L
Serum Procalcitonin	-	1.10	<0.08 ng/mL
Serum Ferritin	2509	9110	21.81-274.66 ng/mL
D-Dimer	-	6600	0-500 ng/mL
Fibrinogen	-	229	180-350 mg/dL
GGT	-	279	15-85 U/Lt
Serum Triglycerides		370	<150 mg/dL

**Table 4:** Iron studies with their reference ranges.

Investigations	Day 2	Reference Range
Serum Iron	12	50-150 microgram/dL
Total Iron Binding Capacity	159	250-450 microgram/dL
Transferrin	7.55%	20-50%
Ferritin	2509	21.81-274.66 ng/mL
Retic count	2.5%	0.5-2.5%

**Table 5:** Special investigations and fever profile were negative. HIV – human immunodeficiency virus, HCV-Ab – antibody against hepatitis-C virus, HbsAg – surface antigen for hepatitis-B virus, ANA Blot – antinuclear antibody blot test, NS1 – nonstructural protein 1, IgM – immunoglobulin M

Investigation	Result	Investigation	Result
HIV (HIV-1, HIV-2, p24Ag)	Non-reactive	Rapid malaria test	Negative
HCV-Ab	Non-reactive	Widal test	Negative
HbSAg	Non-reactive	Blood culture and sensitivity	No growth
Dengue NS1 antigen	Negative	Urine culture and sensitivity	No growth
Leptospira IgM	Negative	Direct/Indirect Coombs test	Negative
ANA Blot	Negative		

For a few days, the patient’s hemoglobin and platelets kept falling without any signs of bleeding. Electrolytes were within normal range, and bilirubin and transaminases kept worsening along with creatinine. With high levels of ferritin, HLH was in consideration as a differential diagnosis, so to establish the diagnosis, we sent further investigations like serum fibrinogen, D-dimer, gamma glutamyl-transferase (GGT), and serum triglycerides (see Table 3). Further scans were done by the third day, as summarized below.

Ultrasonography of the abdomen and pelvis showed bilaterally raised renal cortical echogenicity, an enlarged liver (17 cm), and an enlarged spleen (15 cm). 2D echo showed ejection fraction of 50%, moderate mitral regurgitation, moderate tricuspid regurgitation, mild pulmonary artery hypertension, and grade 1 diastolic dysfunction. Fundoscopy showed grade 1 hypertensive retinopathy in the right eye and grade 2 hypertensive retinopathy in the left eye.

**Figure 1:**
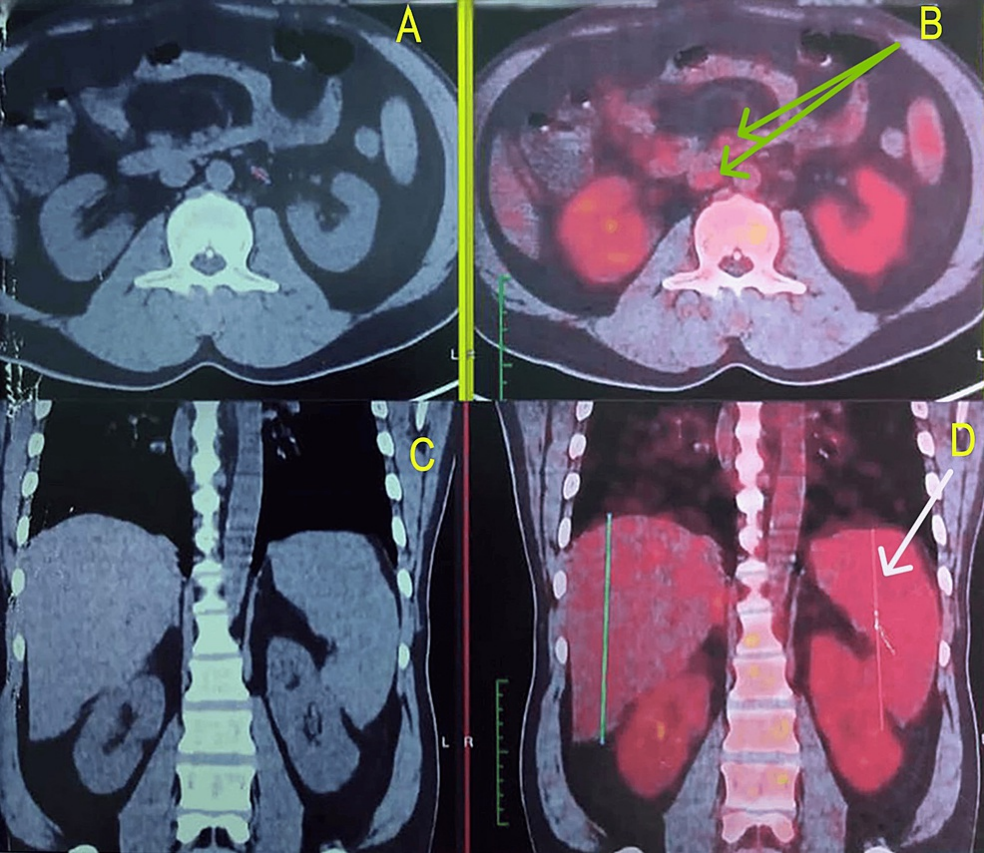
PET-CT scan showing hypermetabolic splenomegaly (see white arrow in image D), heterogenous increased bone marrow uptake, and few weakly metabolic left para-aortic nodes (see green arrows in image B). PET - positron emission tomography (see images B and D), CT - computed tomography (see images A and C)

**Figure 2:**
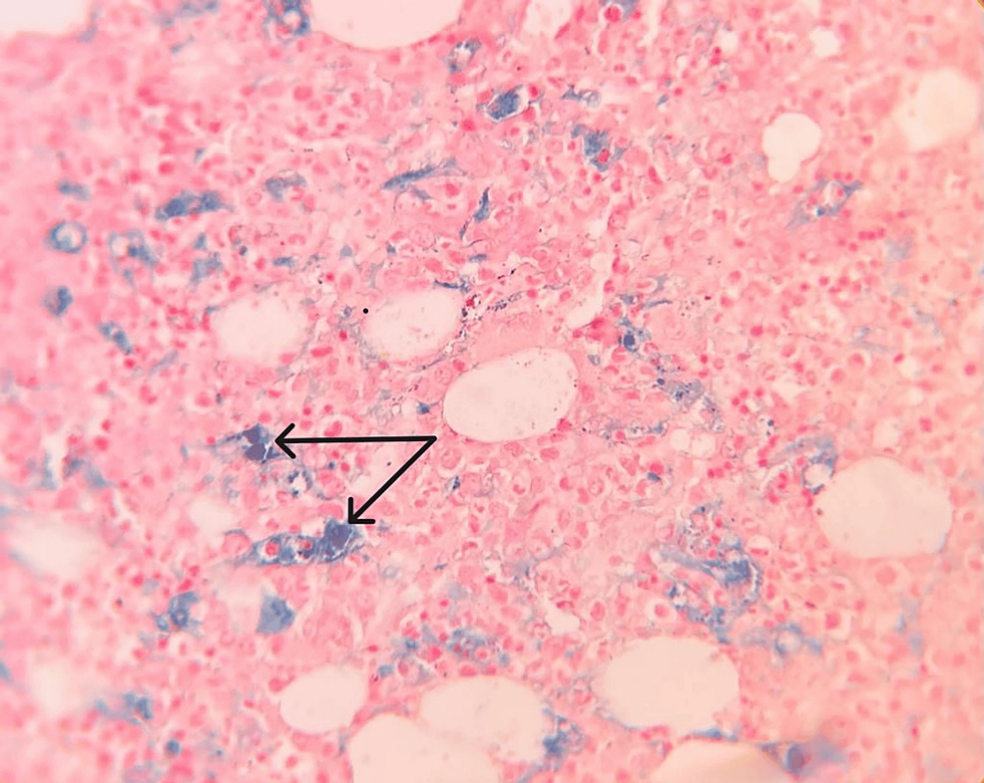
Perl's stain of bone marrow biopsy (400x magnification) showing hemophagocytic histiosis with increased iron stores (Grade 6). Shown with black arrows in the image above.

**Figure 3:**
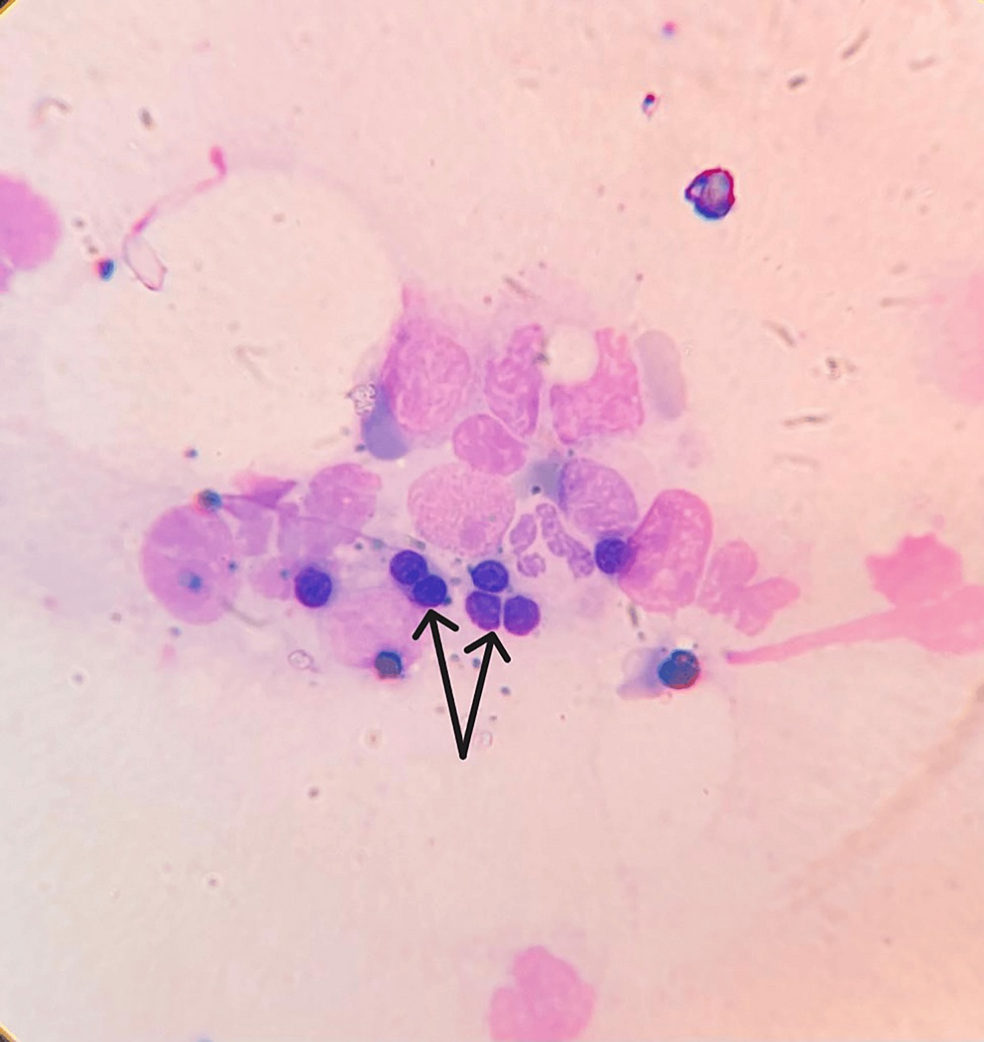
Modified-Giemsa stained bone marrow (40x magnification) shows engulfed red blood cells seen in bone marrow histology slide. Shown with black arrows.

HLH diagnosis was becoming more and more obvious with each test, but no direct correlation was found that could be attributed to trigger HLH. We did a positron emission tomography-computed tomography (PET-CT) to rule out any occult malignancy that could have triggered HLH. PET-CT showed a hypermetabolic spleen with splenomegaly, heterogeneously increased bone marrow uptake, and weakly metabolic left para-aortic lymph nodes (Figure 1). Finally, we had to go for bone marrow aspiration and biopsy under the cover of platelet transfusions and fresh frozen plasma. Bone marrow biopsy showed mildly hypercellular marrow with hemophagocytosis and grade 6 iron stores (Figures 2, 3).

This confirmed our diagnosis of HLH. As per HLH-94 [1], five out of eight criteria have to be positive for an HLH diagnosis. This patient fulfilled six criteria. Natural killer (NK) cell activity and soluble interleukin-2 receptor levels were not sent due to unavailability. The patient was started on inj. etoposide 300 mg twice weekly and inj. dexamethasone 20 mg daily.

Two days later, bone marrow aspiration culture showed the growth of *Pseudomonas aeruginosa*, which was sensitive to piperacillin and tazobactam.

## Discussion

Clinically, HLH presents as a fever with progressive multi-organ dysfunction. Initial presentation varies and can mimic common infections, hepatitis, and encephalitis, or may be labeled as pyrexia of unknown origin (PUO). In HLH, macrophages secrete large amounts of cytokines and cause excessive inflammation that should have been eliminated by NK cells and cytotoxic lymphocytes, but they fail to eliminate these macrophages, resulting in enormous amounts of cluster of differentiation 8 (CD8+) cells, activated macrophages, interferon-gamma, and other cytokines.

In normal individuals, NK cells eliminate damaged, stressed, or infected host cells in a major histocompatibility complex (MHC)- unrestricted manner. MHC class-1 molecules, along with cytotoxic T lymphocytes, lyse macrophages bearing foreign antigens. The elimination of macrophages by these cells happens through perforin-dependent cytotoxicity, which involves immunogenic synapse formation, pore formation in the cell membrane, and delivery of cytolytic granules containing proteases like granzyme B, which causes apoptosis of cell [5,6]. Perforins play an important role in homeostasis after the infective agent has been eliminated. Perforin and FAS-dependent killing of antigen-presenting cells by cytotoxic T-lymphocytes [6] and perforin-dependent killing of activated T-lymphocytes by NK cells [7] are important mechanisms for controlling the immune response. Most of the proteins involved in these processes are encoded by the genes that are defective in familial HLH. Cytokine storm occurs in HLH where interferon-gamma, chemokine ligand-9 (CXCL9), tumor necrosis factor (TNF)-alpha, interleukin-6, interleukin-1, interleukin-12, and soluble interleukin-2 receptor (sIL2R) are produced at extremely high levels leading to multi-organ damage. Expansion of T-lymphocytes leads to splenomegaly; activated lymphocytes and histiocytes infiltrate the liver, causing hepatomegaly, hyperbilirubinemia, and increased liver enzymes [8]. TNF-alpha and interferon-gamma lead to cytopenias, and interleukin release causes fever spikes in a patient.

In 1991 first diagnostic guidelines were published by The Histiocyte Society [9], which were later updated in 1994 as HLH-94 diagnostic criteria had five components, and three more were added in HLH-2004 criteria [10]: fever, cytopenias involving two or more cell lines, hypertriglyceridemia or hypofibrinogenemia, splenomegaly, hemophagocytosis seen in bone marrow, lymph node or spleen, absent or low NK cell activity, raised serum ferritin, elevated sIL2R. HScore is another criterion used in adults for the diagnosis of HLH [11].

The treatment protocol described in HLH-94 is universally followed [1]. Eight weeks of induction therapy for adults with etoposide 150mg/m^2^ IV twice weekly for two weeks, followed by once weekly for the next two weeks. Dose reduction for etoposide should be considered in severe hepatic or renal failure. Along with etoposide, steroids should be given. Dexamethasone is preferred as it crosses the blood-brain barrier, given IV at a dose of 10mg/m^2^ daily for the first two weeks, followed by 5 mg/m^2^ daily for the next two weeks, then 2.5 mg/m^2^ daily for the next two weeks followed by 1.25 mg/m^2^ daily for one week, and then tapered to zero by the eighth week.

HLH-94 also advises starting anti-fungal treatment during dexamethasone initiation. In cases where CNS involvement is not improving with two weeks of therapy, HLH-94 advises intrathecal methotrexate for a maximum of four doses. In addition to HLH-specific therapy, other supportive care like management of anemia, coagulopathy, antibiotics, and monitoring of vitals should be taken care of.

## Conclusions

HLH is a disease that was deadly during the initial years when no proper diagnostic or treatment protocols were available. However, since the availability of etoposide along with dexamethasone, survival chances in this disease have improved drastically. Paired with hematopoietic stem cell transplantation, this is a curable disease. So, the onus is on the treating doctor to quickly identify the disease and start prompt treatment with broad-spectrum empirical antibiotics, anti-fungal drugs, managing bleeding diathesis, maintaining hemodynamics, and monitoring vitals along with HLH-specific therapies. Whenever a diagnosis of HLH is suspected, the treating physician should promptly do additional tests to confirm or rule out HLH. The earlier the HLH-specific treatment is started, the better the prognosis.
